# Binary-Synaptic Plasticity in Ambipolar Ni-Silicide Schottky Barrier Poly-Si Thin Film Transistors Using Chitosan Electric Double Layer

**DOI:** 10.3390/nano12173063

**Published:** 2022-09-03

**Authors:** Ki-Woong Park, Won-Ju Cho

**Affiliations:** Department of Electronic Materials Engineering, Kwangwoon University, 447-1 Wolgye-Dong, Nowon-Gu, Seoul 139-701, Korea

**Keywords:** Ni-silicide, ambipolar channel, polycrystalline silicon, thin-film transistor, artificial synaptic transistor, chitosan electrolyte

## Abstract

We propose an ambipolar chitosan synaptic transistor that effectively responds to binary neuroplasticity. We fabricated the synaptic transistors by applying a chitosan electric double layer (EDL) to the gate insulator of the excimer laser annealed polycrystalline silicon (poly-Si) thin-film transistor (TFT) with Ni-silicide (NiSi) Schottky-barrier source/drain (S/D) junction. The undoped poly-Si channel and the NiSi S/D contact allowed conduction by electrons and holes, resulting in artificial synaptic behavior in both p-type and n-type regions. A slow polarization reaction by the mobile ions such as anions (CH_3_COO^−^ and OH^−^) and cations (H^+^) in the chitosan EDL induced hysteresis window in the transfer characteristics of the ambipolar TFTs. We demonstrated the excitatory post-synaptic current modulations and stable conductance modulation through repetitive potentiation and depression pulse. We expect the proposed ambipolar chitosan synaptic transistor that responds effectively to both positive and negative stimulation signals to provide more complex information process versatility for bio-inspired neuromorphic computing systems.

## 1. Introduction

In recent times, artificial intelligence (AI) technology is being used in everyday life in various fields, such as autonomous cars, intelligent robots, and wearable smart devices [1,2,3]. Processing massive data from numerous variables is essential for AI technology. Although extensive data have been processed on the existing von Neumann-structured CPU chips, the serial method cause a bottleneck between the process and memory. Various attempts have been made to minimize this inefficiency of AI process-specialized circuits using graphics processing units, field-programmable gate arrays, and application-specific integrated circuits [4,5,6]. However, the von Neumann process structure has evident limitations. To overcome these limitations, neuromorphic semiconductors mimicking efficient information processing of the human brain are in the spotlight. The human brain processes extensive information more efficiently and quickly than any other system. Its super-parallel structure of approximately 100 billion neurons and 100 trillion synapses connects them in all directions. When we see, hear, feel, or think about something, neurons exchange information with other neurons by forming electrical spikes. Because of the parallel structure, the human brain processes information with an energy consumption of 20 W and has no bottleneck inefficiency [7,8]. In the early research, a two-terminal memristor attracted attention as a synaptic device with a structure and mechanism similar to these biological synapses. However, typical two-terminal memristors based on conducting filaments or phase change materials have the disadvantage of requiring high write/read currents due to the high conductance, resulting in excessive power loss as the array scale increases [9]. Accordingly, a three-terminal synaptic device based on a transistor structure with an added gate is also being actively studied.

Particularly, electrolyte-based synaptic transistors can mimic ion-dependent signaling such as K^+^, Na^+^, and Cl^−^ in biological synapses [10,11,12]. One of the primary advantages of electrolyte-based transistors is low driving voltage. Compared to conventional insulating materials (e.g., 300-nm-thick SiO_2_ is ~10 nF/cm^2^), the electrolyte has a significantly large capacitance (~1 μF/cm^2^); therefore, the driving voltage of the transistor can be drastically reduced [13,14].

Chitosan is an excellent biocompatible polymer for the synaptic transistor electrolyte layer and is purified from chitin, the second most abundant biopolymer on earth. Owing to the electric double layer (EDL) effect of chitosan electrolyte, high gate capacitance (>1.0 μF/cm^2^) can be easily obtained from high-density mobile ions, enabling synaptic behavior [15,16,17]. Because chitosan is an organic biomaterial, it has chemical/mechanical weaknesses, and this limits various processes or structures of synaptic transistors. In previous studies, we overcame the limitation by stacking biocompatible high-k Ta_2_O_5_ film as a chitosan barrier layer, enabling a lithographic process to be applied to various structures [18,19]. Furthermore, the previously reported chitosan-related synaptic transistors exhibited unipolar operation characteristics of n-channel operation [20,21,22], which is insufficient to provide more complex information processing diversity in neural processing. The biological synapse releases the excitatory or inhibitory neurotransmitters for processing the signals, resulting in an excitatory or inhibitory post-synaptic potential [23,24]. Such two types of synaptic responses can be reconfigured between different responses by external environment or emotion [25]. In other words, an excitatory or inhibitory response can occur even with the same stimuli depending on the current situation. However, the reconfiguration of synaptic responses is difficult to achieve in the unipolar synaptic transistors [26].

Here, we propose an ambipolar chitosan synaptic transistor that effectively responds to binary neuroplasticity. We use excimer laser annealed (ELA) polycrystalline silicon (poly-Si) as the channel layer for the ambipolar operation. In addition, we apply an isolated top-gated transistor structure and a Ni-silicide (NiSi) Schottky barrier source/drain (S/D) that minimizes contact resistance optimized for nanoscale process and complex connection [27]. Because of the ambipolar undoped poly-Si channel, artificial synaptic behavior has been realized in both p- and n-regions via holes and electrons, respectively. We demonstrate the excitatory post-synaptic current (EPSC) modulations and stable conductance modulation through repetitive potentiation/depression pulses. We expect the proposed ambipolar chitosan synaptic transistor that responds effectively to positive and negative stimulation signals to secure functionalities for the bio-inspired neuromorphic computing system.

## 2. Experimental Details

### 2.1. Device Fabrication

**Figure 1:**
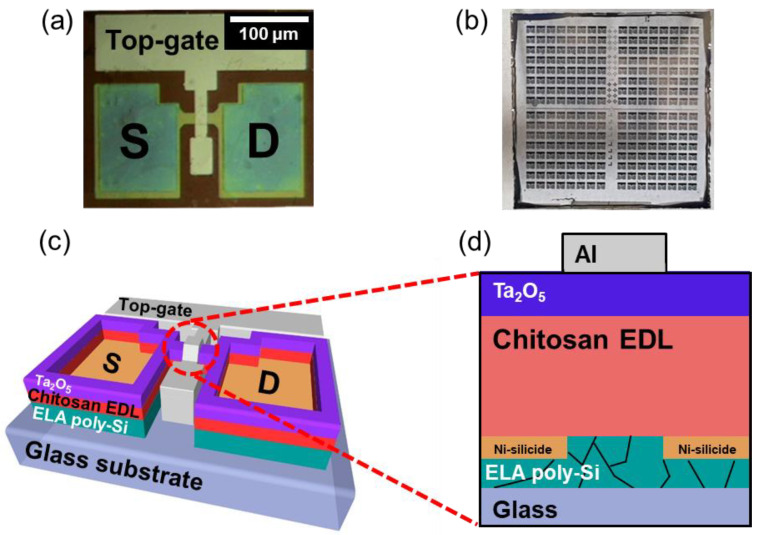
(**a**) Optical microscopic image of the ambipolar chitosan synaptic transistor with Ni-silicide (NiSi) source/drain (S/D) and (**b**) photographic array images of the ELA glass substrate. Schematics of the (**c**) three-dimensional and (**d**) cross-sectional structure of the synaptic transistor.

We fabricated the top-gate structure ambipolar chitosan synaptic transistor on a glass substrate with a 160-nm-thick ELA crystallized poly-Si film. For the channel layer (post-synapse), we defined the active channel area with width/length = 20/10 μm by photolithography; we wet etched it using silicon etchant. For NiSi S/D junctions, we deposited an 80-nm-thick Ni film with an E-beam evaporator. For the formation of NiSi, we performed the 2.45 GHz frequency of microwave annealing (MWA) process at 600 W (corresponding to approximately 400 °C thermal temperature [28,29]) in N_2_ ambient air for 2 min. We removed unreacted Ni using a mixture of H_2_SO_4_:H_2_O_2_ = 1:1. We formed the chitosan electrolyte EDL (neurotransmitter) using the following procedure. We prepared the chitosan electrolyte solution using a 2 wt% chitosan powder (deacetylation degree > 75%) dissolved in 2 wt% acetic acids; we spin-coated the chitosan EDL layer, dried it in ambient air for 24 h, and oven-baked it at 130 °C for 10 min. The thickness of the chitosan EDL was 130 nm (±5 nm deviation). Subsequently, we deposited a high-*k* Ta_2_O_5_ dielectric layer with 80 nm thickness using RF magnetron sputtering as a chemical/mechanical reinforcing barrier layer of the organic chitosan electrolyte film. For the top-gate electrode (pre-synapse), we deposited a 150-nm-thick Al film using an e-beam evaporator and then formed it using a lift-off method. Finally, we opened the S/D contact hole for electrical measurement using a reactive ion etching process. Figure 1a shows a 300-times-magnified microscopic image of the ambipolar chitosan synaptic transistor with NiSi S/D, and Figure 1b shows the synaptic transistors array on an ELA glass substrate. Due to the Ta_2_O_5_ barrier layer on the chitosan electrolyte, the ambipolar chitosan synaptic transistor could be patterned by photolithography.

### 2.2. Characterization and Measurements

We analyzed the optical microscopic image of the fabricated ambipolar chitosan synaptic transistor using an SV−55 Microscope System (SOMETECH, Seoul, Korea). We measured the Fourier transform infrared (FT-IR) spectroscopy analysis of chitosan electrolyte EDL film using IFS 66v/S and HYPERION 3000 ALPHA FT-IR microscope (Bruker Optics, Billerica, MA, USA). We measured the transfer, output characteristics, and synaptic behavior using an Agilent 4156B Precision Semiconductor Parameter Analyzer (Hewlett-Packard Co., Palo Alto, Santa Clara, CA, USA). To apply a pre-synapse spike, we applied electrical pulses using Agilent 8110A Pulse Generator (Hewlett-Packard Co., Palo Alto, Santa Clara, CA, USA). We evaluated the crystal structure of the microwave annealed NiSi film through X-ray diffraction analysis using a SmartLab X-ray diffractometer (Rigaku Co., Tokyo, Japan).

## 3. Results and Discussion

### 3.1. Device Structure of Ambipolar Chitosan Synaptic Transistor

Recent studies on synaptic transistors using chitosan electrolytes as EDL have restricted the options in the device fabrication process due to limitations of the mechanical/chemical weakness of the organic chitosan layer. Representatively, the patterning process using a shadow mask that does not require additional etching and cleaning has been forced after the formation of the chitosan electrolyte layer [30,31,32,33,34]. However, through our latest research, we secured the possibility of applying the lithography patterning process by laminating the high-k Ta_2_O_5_ as a barrier layer [18,19]. Subsequently, as the fabrication process limit expanded, we applied ambipolar-type undoped poly-Si as a channel layer and MWA-silicided Ni S/D to develop an advanced-structure chitosan synaptic transistor with a photolithography process optimized for high integration. The Si-based ambipolar synaptic transistor in our study can provide CMOS process compatibility and high stability compared to other channel materials such as oxide semiconductor or graphene. The schematic structure of the fabricated top-gate type chitosan synaptic transistor is shown in Figure 1c,d.

### 3.2. Characterization of Chitosan Electrolyte EDL and NiSi S/D

**Figure 2:**
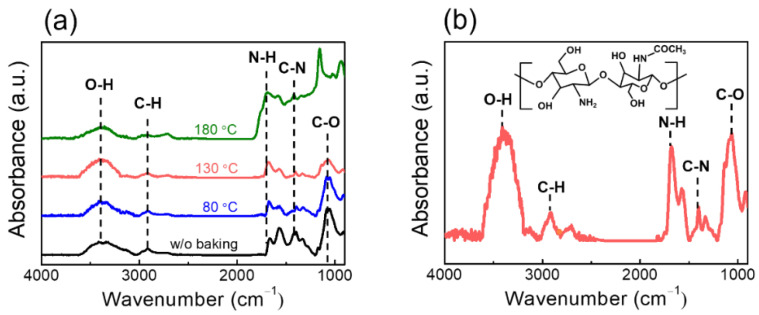
(**a**) Fourier transform infrared spectroscopy (FT-IR) of the chitosan electrolyte film with varying baking temperature and (**b**) 130 °C baking condition. Inset: Molecular structure of the chitosan electrolyte.

We applied the chitosan electrolyte EDL as a neurotransmitter in the biologic synapse to implement synaptic behavior. For fabricating a photolithography-processed chitosan synaptic transistor, an appropriate chitosan oven baking must be applied to prevent the swelling/outgassing of the chitosan electrolyte film due to chemical/thermal damage during the photolithography process [18]. Figure 2a shows the FT-IR spectra of the chitosan electrolyte film according to the oven baking condition. The typical chitosan FT-IR peak shape remains stable until the 130 °C oven baking. Figure 2b shows the detailed FT-IR spectra at 130 °C for accurate band characterization of 130 °C oven-baked chitosan films. The band around 3412 cm^−1^ is attributed to the O-H peak. The C-H stretching peak is around 2902 cm^−1^. In addition, the band at 1672 cm^−1^ is ascribed to the N-H bending of -NH_2_. The bands at 1398 cm^−1^ are ascribed to the C-N (amide) peak. The C-O peak appears at 1066 cm^−1^. These bands are primarily reported in synaptic transistor studies using chitosan electrolytes as EDL [35,36]. Therefore, we prevented chemical/thermal damage during the photolithography process through 130 °C oven baking in which the band of chitosan electrolyte film remained stable.

**Figure 3:**
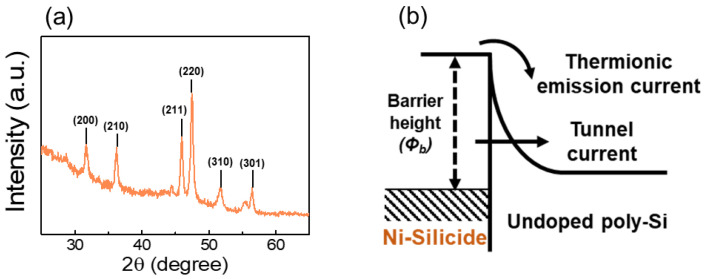
(**a**) X-ray diffraction pattern of NiSi S/D film formed by 600 W of microwave irradiation annealing. (**b**) Schematic band diagram of NiSi and poly-Si interface.

We applied NiSi as an S/D electrode on the undoped poly-Si channel to provide interface stability and low contact resistance compared to metal-Si junctions by forming a Schottky barrier. As a self-aligned silicide annealing process of NiSi, we performed a short-time efficient MWA—a unique volumetric direct heating method promising to achieve advanced Si CMOS process [37]. Figure 3a,b shows the XRD pattern of NiSi formed by 600 W of MWA treatment and the Schottky contact diagram between NiSi and Si interface, respectively. The XRD peaks appearing in the diffraction pattern were referred to as the peak data of the International Committee for Diffraction Data powder diffraction file for accurate identification [38]. Certain (200), (210), (211), (220), (310), and (301) peaks indicating the preferentially oriented peaks of NiSi were identified. The NiSi provides strong immunity to the short channel effect and bridging failure, which can be fatal for scaled neural-ICs resulting from a short-circuit area between the gate and S/D [39,40].

### 3.3. Electrical Properties of the Ambipolar Chitosan Synaptic Transistor

**Figure 4:**
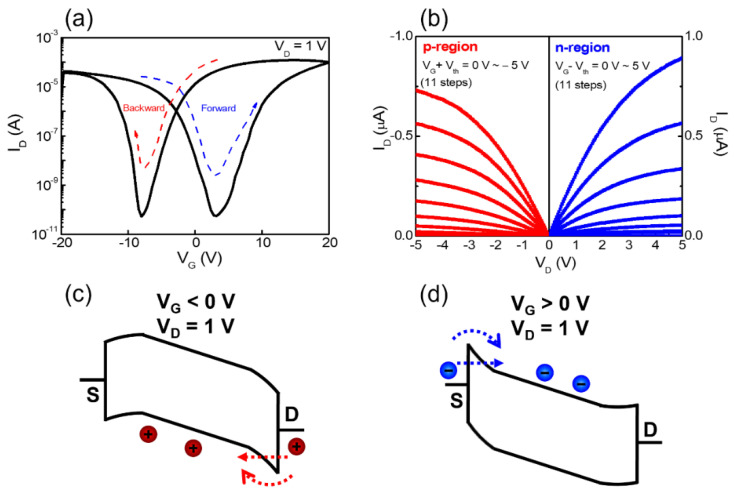
(**a**) Double-sweep transfer (I_D_−V_G_) curves swept from forward to backward at V_D_ = 1 V and (**b**) output curves (I_D_−V_D_) in each of the p- and n-region. Current flow mechanism in (**c**) p-region and (**d**) n-region, respectively.

Figure 4a shows the double-sweep transfer characteristic (I_D_–V_G_) curves of the ambipolar chitosan synaptic transistor at a constant drain voltage (V_D_) of 1 V. The gate voltage sweep was swept forward from −20 V to 20 V and then swept backward from 20 V to −20 V. Due to the undoped ambipolar type poly-Si channel, p- and n-type operate well by hole and electron accumulation, respectively. Meanwhile, the normally off behavior is attributed to carrier depletion caused by the NiSi Schottky barriers [41]. Figure 4c,d show this current flow mechanism. When the negative gate bias is applied simultaneously with drain bias, the holes are injected into the poly-Si channel according to the mechanism of tunneling or thermionic emission. Likewise, in the case of positive bias applied to the gate, the electrons are injected into the channel. Meanwhile, a wide hysteresis window appears between forward and reverse sweeps under double-sweep transfer conditions. It is due to the slow polarization reaction of mobile ions in the chitosan electrolyte EDL. Figure 4b shows the symmetric output characteristics (I_D_-V_D_) curves measured in the p- and n-regions. Ambipolar-type channel transistors typically exhibit leakage currents on output curves due to opposite carriers (electrons in p-region, holes in n-region) at high V_D_ [42,43,44]. However, in the low V_D_ required for synaptic operation (less than 5 V), such leakage current does not significantly affect and saturate stably.

**Figure 5:**
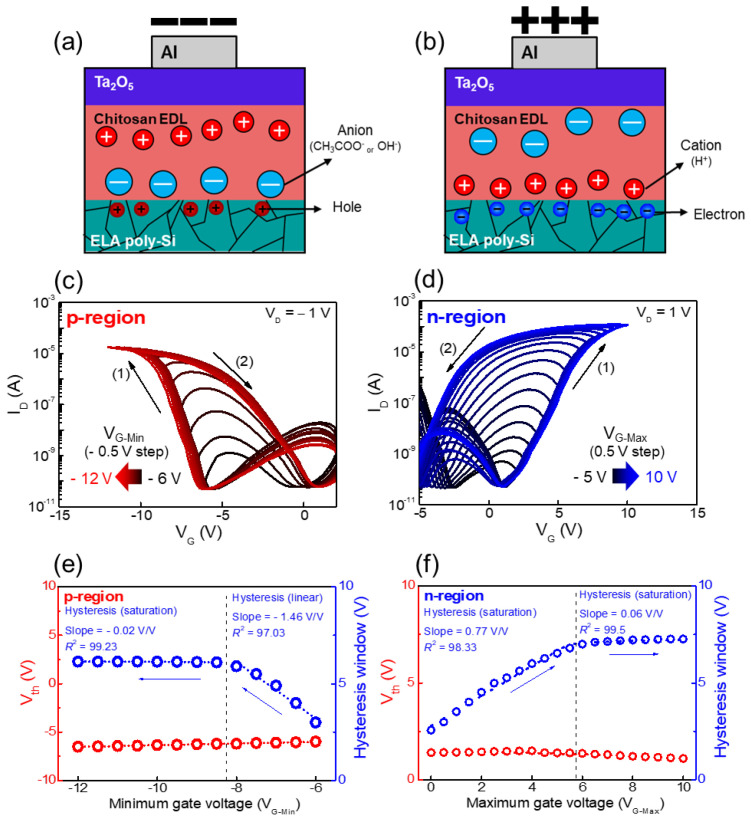
Mobile ion migration mechanism in the chitosan EDL of ambipolar chitosan synaptic transistor according to the (**a**) negative and (**b**) positive gate bias conditions. (**c**) p-region double-sweep transfer (I_D_−V_G_) curves according to V_G_min_ (−6 to −12 V in −0.5 V decrements). (**d**) n-region double-sweep transfer (I_D_−V_G_) curves according to V_G_max_ (−5 to 10 V in 0.5 V increments). V_th_ and hysteresis window variation corresponding to (**e**) V_G-min_ at p-region and (**f**) V_G_max_ at n-region.

Figure 5a,b illustrate the operating mechanism of the ambipolar chitosan synaptic transistor according to the negative and positive gate bias conditions, respectively. Most of the cations (H^+^) are in the chitosan electrolyte EDL; however, anions (CH_3_COO^−^ or OH^−^) are also present [30,45]. When a bias is applied to the top gate, these mobile ions cause a slow polarization reaction in the chitosan electrolyte EDL. Under the negative bias condition, anions accumulate at the interface of the ambipolar-type poly-Si channel. Under the positive bias condition, cations accumulate at the channel interface. Figure 5c,d show a double-sweep transfer curve measured separately for p- and n-regions, respectively. In the p-region, holes in the ambipolar poly-Si channel act as major carriers. In the n-region, electrons act as major carriers. First, the p-region double-sweep transfer curve in Figure 5c was measured by decreasing the minimum gate voltage (V_G-Min_) sweep range from −6 V to −12 V (in −0.5 V steps). The clockwise hysteresis in the curves occurred due to negative bias applied to the gate during the double sweep, which is observed in a p-type operating EDL synaptic transistor [46,47]. Figure 5e shows the hysteresis window and the threshold voltage (V_th_) variation according to the V_G-Min_ on the p-region. As the V_G-Min_ decreases, the V_th_ remains fixed, and the hysteresis window linearly increases and then saturates. This is because more anions (CH_3_COO^−^ or OH^−^) accumulate at the channel interface by the strong negative bias and require a strong positive bias to diffuse back. Then, after enough anions are accumulated, the hysteresis window remains saturated. Second, the opposite polarity of the n-region double-sweep transfer curve in Figure 5d was measured by increasing the maximum gate voltage (V_G-Max_) sweep range from −5 V to 10 V (in 0.5 V steps). Contrary to the p-region, anti-clockwise hysteresis occurred at the n-region due to positive bias applied to the gate during the double sweep, which is observed in an n-type operating EDL synaptic transistor [48,49]. Figure 5f shows the hysteresis window and the V_th_ variation according to the V_G-Max_ on the n-region. Considering the case of the p-region, the increasing tendency of the hysteresis window in the n-region is due to the accumulation of cations (H^+^). Consequently, we examined the fundamental operation of the ambipolar chitosan synaptic transistor due to the slow mobile ion polarization in the chitosan.

### 3.4. Synapse Mimicking Properties of the Ambipolar Chitosan Synaptic Transistor

**Figure 6:**
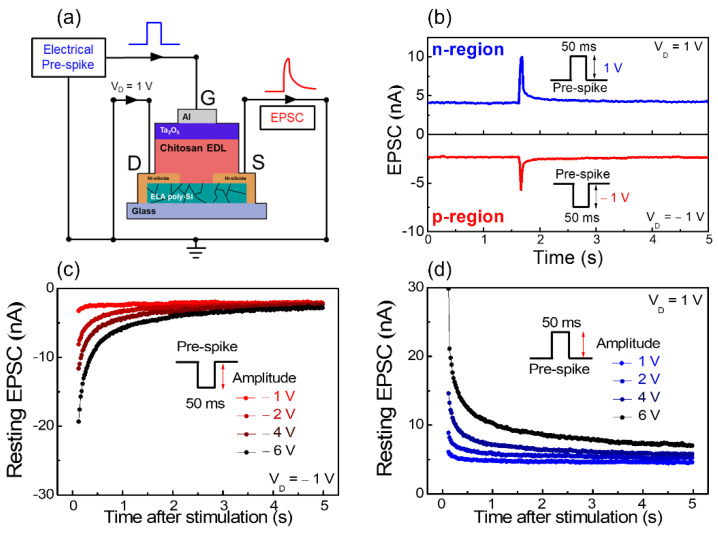
(**a**) Schematic of the EPSC measurement circuit of the ambipolar chitosan synaptic transistor. (**b**) EPSC retention characteristics due to pre-synapse spike in both regions. Resting EPSC variation for pre-synapse spike amplitude of the (**c**) p-region and (**d**) n-region.

The synaptic behavior of synaptic transistor operation is essential to mimic the biologic synapse functionality and mechanism. In biology, neurons and synapses behave like the two fundamental computational engines in the human brain. The signal spikes generated by pre-synaptic neurons are transmitted to post-synaptic neurons through neurotransmitters [50]. In the fabricated ambipolar chitosan synaptic transistor, the electrical spikes applied at the Al top-gate (pre-synapse) migrate the mobile ions (neurotransmitter) in the Ta_2_O_5_/chitosan EDL composite insulator layer (synaptic cleft) to the ambipolar-type poly-Si channel (post-synapse). Consequently, electrical spikes cause excitatory current in the post-synapse channel, which is called excitatory postsynaptic current (EPSC). EPSC is a fundamental representation of synaptic strength [51]. Figure 6a shows the simplified schematic of the EPSC measurement. Figure 6b shows the EPSC retention characteristics for a single pre-synapse spike in the n- and p-regions. For n-region measurement, a pre-synapse spike with an amplitude of 1 V and duration of 50 ms was applied under a constant V_D_ of 1 V. For p-region measurement, a pre-synapse spike with an amplitude of −1 V and duration of 50 ms was applied under a constant V_D_ of −1 V. After the pre-synapse spike, the EPSC rises to a peak and then slowly decreases by the slow polarization reaction of the mobile ions in the chitosan EDL. This tendency is well-implemented in both n- and p-regions. These temporal profiles of the EPSC are similar to biological excitatory synapses. Just a few seconds of the EPSC retention time scale implies short-term synaptic plasticity, which allows the basis of learning and memory of the nervous information process system [20]. Figure 6c,d show the resting EPSC after stimulation by an amplitude-variated pre-synapse spike in the p- and n-regions, respectively. After 5 s of spike stimulation, the resting EPSC absolute value slightly increased from −2.0 nA to −4.1 nA as the pre-synapse spike amplitude increased from −1 V to −6 V in the p-region. In the n-region, as the pre-synapse spike amplitude increased from 1 to 6 V, the resting EPSC absolute value slightly increased from 4.6 nA to 7 nA. Through the resting EPSC tendency, we found that increasing the amplitude of the pre-synapse spike can affect the temporal property of the synaptic plasticity in both p- and n-regions.

**Figure 7:**
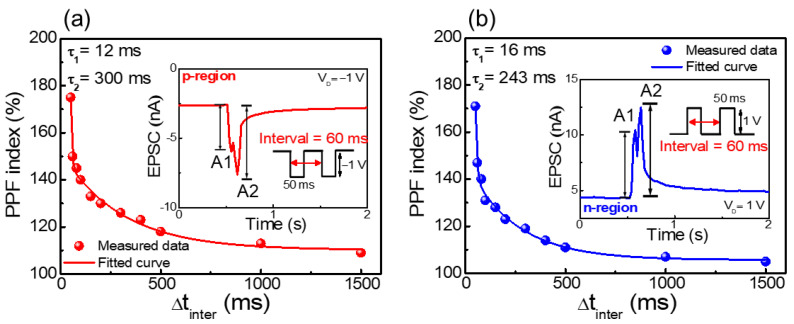
Paired-pulse facilitation (PPF) index (defined as A2/A1 × 100%) of the (**a**) p-region and (**b**) n-region. The solid line represents the fitting curve of the double-phase exponential function. Inset: EPSC triggered by a paired spike with a 60 ms interval.

As a form of short-term synaptic plasticity, the paired-pulse facilitation (PPF) characteristic represents the dynamic enhancement of neurotransmitters in biological neural synapses, involved in several neural tasks such as simple learning and encoding temporal information [52]. PPF is the degree of facilitation between the first and second pre-synapse spikes. In the fabricated ambipolar chitosan synaptic transistor, it is possible to know the degree to which mobile ions moved to the channel interface by the first pre-synapse spike, further facilitated by the second spike before diffusing back. Figure 7a,b show the PPF index in the p- and n-regions, respectively. The PPF index can be obtained as a ratio of the EPSC value (A1) triggered by the first spike and the EPSC value (A2) triggered by the second spike. For extracting the PPF, two consecutive spikes with a duration of 50 ms (p-region amplitude = −1 V, n-region amplitude = 1 V) were applied by adjusting the interval time (Δt_inter_) between spikes. The shorter the interval between the two spikes, the more the occurrence of EPSC facilitation. At 50 ms intervals, the PPF index increased to 175% in the p-region and 171% in the n-region. The decay of the PPF index with the spike interval was fitted by a double exponential decay function, the extracted relaxation time constants are τ_1_ = 12 ms, τ_2_ = 300 ms in the p-region, and τ_1_ = 16 ms, τ_2_ = 243 ms in the n-region, respectively. In the biological synapses, τ_1_ is about tens of milliseconds (rapid phase) and τ_2_ is about hundreds of milliseconds (slow phase), which is similar to the extracted values in this study [53,54].

**Figure 8:**
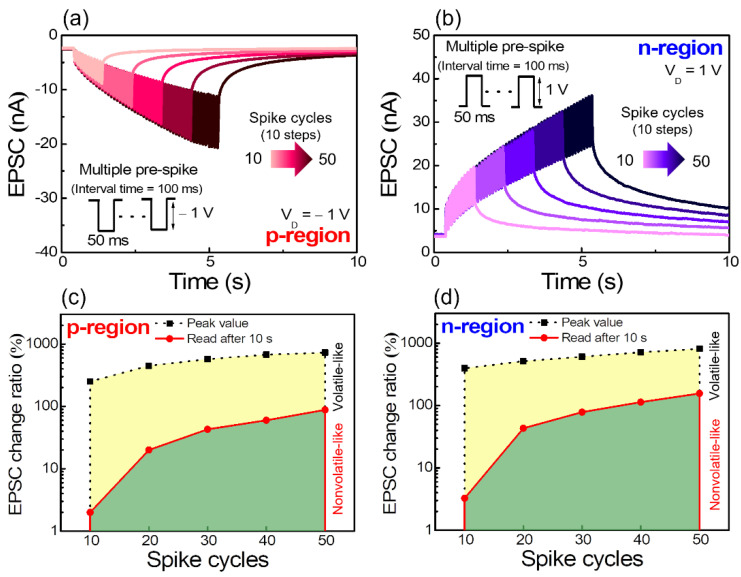
Dynamic EPSC retention characteristics in response to multiple pre-synapse spikes (10, 20, 30, 40, and 50 cycles) of the (**a**) p-region and (**b**) n-region. Spike cycle dependence of the EPSC change ratio at the (**c**) p-region and (**d**) n-region.

Synaptic plasticity can be gradually enhanced by multiple pre-synaptic spikes. We accomplished actual information processing between synapses through multiple pre-synaptic spikes. Figure 8a,b show EPSC responses to multiple pre-synapse spikes in the p- and n-regions, respectively. In the single pre-synapse spike condition (duration = 50 ms, p-region amplitude = −1 V, and n-region amplitude = 1 V) applied thus far, spikes were continuously applied from 10 to 50 times with a spike interval time of 100 ms. The more the multiple pre-synapse spikes are applied, the more the facilitation of peak EPSC and increase in the resting EPSC. Figure 8c,d show spike cycle dependence of the EPSC change ratio in the p- and n-regions. EPSC change ratio means ((I − I_0_)/I_0_) × 100%, where I_0_ and I are the channel current before and after the gate spike stimulation, respectively [9]. The EPSC change ratio after 10 s increases significantly with the increase in the cycles of pulse, indicating a trend from short-term to long-term synaptic plasticity. Thus, we successfully confirmed the synaptic plasticity in both p- and n-regions by accumulating the mobile ions in the chitosan electrolyte EDL to poly-Si channel on the fabricated ambipolar chitosan synaptic transistor.

**Figure 9:**
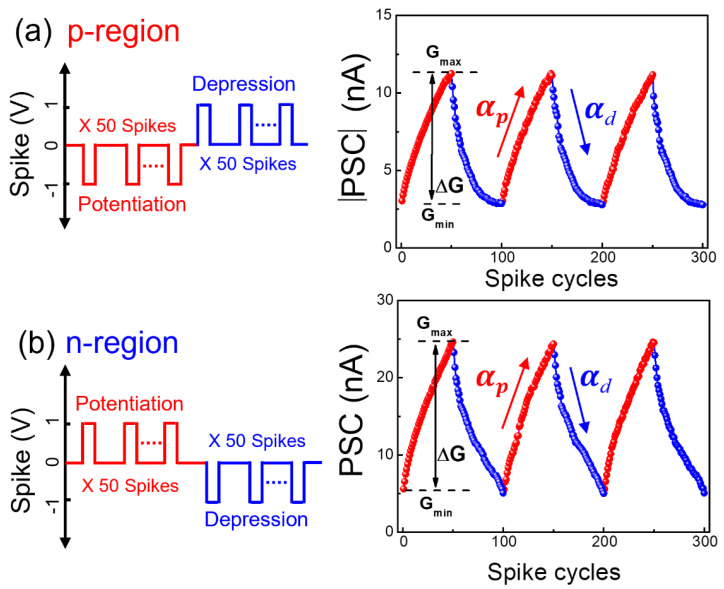
Synaptic weight update states characterized by the gradual potentiation/depression curves for the (**a**) p- and (**b**) n-regions.

To construct a practical neuromorphic system using the conductance variability of the ambipolar chitosan synaptic transistor, we investigated the potentiation and depression curves. When the potentiation/depression properties of the ambipolar chitosan synaptic transistors are applied to an artificial neural network (ANN), more diverse functionalities can be secured to the bio-inspired neuromorphic computing systems [55]. Figure 9a,b show the synaptic weight update states according to 50 excitatory/inhibitory pre-synapse spike cycles in the p- and n-regions, respectively. The synaptic weight was gradually changed by each excitatory/inhibitory stimulus. We extracted weight update margins (ΔG = G_max_ − G_min_, G refers to the channel conductance) and nonlinearity (α_p_ and α_d_, the ideal value = 1) from the potentiation/depression curves for both p- and n-regions [56]. In the learning process implemented through ANN, each of these ΔG values are involved in the efficiency of unidirectional learning for both p- and n-regions. The ΔG values in the p- and n-regions are identified as 15.42 and 24.69, respectively. In addition, the values of α_p_ are 1.72 in the p-region and 1.69 in the n-region. The values of α_d_ are −1.63 in the p-region and −0.41 in the n-region, respectively, demonstrating a better learning efficiency in the n-region. In particular, even with the same stimulus (positive or negative spike), the synaptic weight can be potentiated or depressed by whether the ambipolar synaptic transistor operates in the n-region or the p-region. This property can more closely mimic the central nervous system of a human brain, which reacts differently to the same stimulus depending on the external environment [25]. Accordingly, the ambipolar synaptic behavior not only enables various information processing for positive and negative signals using both n- and p-regions but is also more suitable for mimicking the biological human brain.

## 4. Conclusions

We proposed the ambipolar chitosan synaptic transistor securing complex information process versatility in neural processing. We laminated high-k Ta_2_O_5_ on chitosan EDL as a barrier layer to enable a photolithography process of ambipolar chitosan synaptic transistor. Binary synaptic operation in both the p-region and n-region was successfully implemented by applying ambipolar type poly-Si channel and slow polarization reaction of chitosan mobile ions (H^+^, CH_3_COO^−^, and OH^−^). We demonstrated synaptic properties, including EPSC and gradual potentiation/depression characteristics, in the ambipolar chitosan synaptic transistor in each of the p- and n-regions. The fabricated devices can mimic stably the biological synaptic properties. Furthermore, depending on whether the device operates in the n- or p-region, the artificial synapse is potentiated or depressed even under the same gate bias stimulus. The combination of such binary synaptic properties of ambipolar chitosan synaptic transistor provides the capacity to imitate more complex functions in the biological neural system.

## Data Availability

Not applicable.

## References

[1] Tavakoli M., Carriere J., Torabi A. (2020). Robotics, smart wearable technologies, and autonomous intelligent systems for healthcare during the COVID-19 pandemic: An analysis of the state of the art and future vision. Adv. Intell. Syst..

[2] He W., Li Z., Chen C.P. (2017). A survey of human-centered intelligent robots: Issues and challenges. IEEE/CAA J. Autom. Sin..

[3] Li B.H., Hou B.C., Yu W.T., Lu X.B., Yang C.W. (2017). Applications of artificial intelligence in intelligent manufacturing: A review. Front. Inf. Technol. Electron. Eng..

[4] Nurvitadhi E., Sheffield D., Sim J., Mishra A., Venkatesh G., Marr D. Accelerating Binarized Neural Networks: Comparison of FPGA, CPU, GPU, and ASIC. Proceedings of the 2016 International Conference on Field-Programmable Technology (FPT).

[5] Nurvitadhi E., Venkatesh G., Sim J., Marr D., Huang R., Ong Gee Hock J., Liew Y.T., Srivatsan K., Moss D., Subhaschandra S. Can FPGAs Beat GPUs in Accelerating Next-Generation Deep Neural Networks?. Proceedings of the 2017 ACM/SIGDA International Symposium on Field-Programmable Gate Arrays.

[6] Greenberg-Toledo T., Mazor R., Haj-Ali A., Kvatinsky S. (2019). Supporting the momentum training algorithm using a memristor-based synapse. IEEE Trans. Circuits Syst. I Regul. Pap..

[7] Shi J., Ha S.D., Zhou Y., Schoofs F., Ramanathan S. (2013). A correlated nickelate synaptic transistor. Nat. Commun..

[8] Zhu L.Q., Wan C.J., Guo L.Q., Shi Y., Wan Q. (2014). Artificial synapse network on inorganic proton conductor for neuromorphic systems. Nat. Commun..

[9] Yang C.S., Shang D.S., Liu N., Fuller E.J., Agrawal S., Talin A.A., Li Y.Q., Shen B.G., Sun Y. (2018). All-solid-state synaptic transistor with ultralow conductance for neuromorphic computing. Adv. Funct. Mater..

[10] Zhu L.Q., Wan C.J., Gao P.Q., Liu Y.H., Xiao H., Ye J.C., Wan Q. (2016). Flexible proton-gated oxide synaptic transistors on Si membrane. ACS Appl. Mater. Interfaces.

[11] Shao F., Yang Y., Zhu L.Q., Feng P., Wan Q. (2016). Oxide-based synaptic transistors gated by sol–gel silica electrolytes. ACS Appl. Mater. Interfaces.

[12] Kalia M., Meijer H.G., van Gils S.A., van Putten M.J., Rose C.R. (2021). Ion dynamics at the energy-deprived tripartite synapse. PLoS Comput. Biol..

[13] Lee J., Panzer M.J., He Y., Lodge T.P., Frisbie C.D. (2007). Ion gel gated polymer thin-film transistors. J. Am. Chem. Soc..

[14] Choi J.H., Xie W., Gu Y., Frisbie C.D., Lodge T.P. (2015). Single ion conducting, polymerized ionic liquid triblock copolymer films: High capacitance electrolyte gates for n-type transistors. ACS Appl. Mater. Interfaces.

[15] Hosseini N.R., Lee J.S. (2015). Biocompatible and flexible chitosan-based resistive switching memory with magnesium electrodes. Adv. Funct. Mater..

[16] Liu Y.H., Zhu L.Q., Feng P., Shi Y., Wan Q. (2015). Freestanding artificial synapses based on laterally proton-coupled transistors on chitosan membranes. Adv. Mater..

[17] Fu W.H., Li J., Jiang D.L., Yang Y.H., Chen Q., Zhu W.Q., Zhang J.H. (2020). Proton conducting C_3_N_4_/chitosan composite electrolytes based InZnO thin film transistor for artificial synapse. Org. Electron..

[18] Kim S.H., Cho W.J. (2021). Lithography processable Ta_2_O_5_ barrier-layered chitosan electric double layer synaptic transistors. Int. J. Mol. Sci..

[19] Min S.Y., Cho W.J. (2020). CMOS-compatible synaptic transistor gated by chitosan electrolyte-Ta_2_O_5_ hybrid electric double layer. Sci. Rep..

[20] Hu W., Jiang J., Xie D., Liu B., Yang J., He J. (2019). Proton–electron-coupled MoS_2_ synaptic transistors with a natural renewable biopolymer neurotransmitter for brain-inspired neuromorphic learning. J. Mater. Chem. C.

[21] Yu F., Zhu L.Q., Gao W.T., Fu Y.M., Xiao H., Tao J., Zhou J.M. (2018). Chitosan-based polysaccharide-gated flexible indium tin oxide synaptic transistor with learning abilities. ACS Appl. Mater. Interfaces.

[22] Liu R., Zhu L.Q., Wang W., Hui X., Liu Z.P., Wan Q. (2016). Biodegradable oxide synaptic transistors gated by a biopolymer electrolyte. J. Mater. Chem. C.

[23] Sundaram R.S., Gowtham L., Nayak B.S. (2012). The role of excitatory neurotransmitter glutamate in brain physiology and pathology. Asian J. Pharm. Clin. Res..

[24] McCormick D.A. (1989). GABA as an inhibitory neurotransmitter in human cerebral cortex. J. Neurophysiol..

[25] Yao Y., Huang X., Peng S., Zhang D., Shi J., Yu G., Liu Q., Jin Z. (2019). Reconfigurable artificial synapses between excitatory and inhibitory modes based on single-gate graphene transistors. Adv. Electron. Mater..

[26] Wang Y., Liao Q., She D., Lv Z., Gong Y., Ding G., Ye W., Chen J., Xiong Z., Wang G. (2020). Modulation of binary neuroplasticity in a heterojunction-based ambipolar transistor. ACS Appl. Mater. Interfaces.

[27] Choi S.J., Han J.W., Kim S., Moon D.I., Jang M., Choi Y.K. (2010). High-performance polycrystalline silicon TFT on the structure of a dopant-segregated schottky-barrier source/drain. IEEE Electron. Device Lett..

[28] Shin J.W., Cho W.J. (2018). Microwave annealing effects of indium-tin-oxide thin films: Comparison with conventional annealing methods. Phys. Status Solidi A.

[29] Kim S.H., Cho W.J. (2021). Improvement of structural, electrical, and optical properties of sol–gel-derived indium–tin-oxide films by high efficiency microwave irradiation. J. Nanosci. Nanotechnol..

[30] Long T.Y., Zhu L.Q., Guo Y.B., Ren Z.Y., Xiao H., Ge Z.Y., Wang L. (2019). Flexible oxide neuromorphic transistors with synaptic learning functions. J. Phys. D Appl. Phys..

[31] Chen C., He Y., Zhu L., Zhu Y., Shi Y., Wan Q. (2021). Flexible dual-date MoS_2_ neuromorphic transistors on freestanding proton-conducting chitosan membranes. IEEE Trans. Electron. Devices.

[32] Ren Z.Y., Zhu L.Q., Yu F., Xiao H., Xiong W., Ge Z.Y. (2019). Synaptic metaplasticity of protonic/electronic coupled oxide neuromorphic transistor. Org. Electron..

[33] Yu F., Zhu L.Q., Xiao H., Gao W.T., Guo Y.B. (2018). Restickable oxide neuromorphic transistors with spike-timing-dependent plasticity and pavlovian associative learning activities. Adv. Funct. Mater..

[34] Lu G., Liu Y., Lin F., Gen K., Wu W., Yao R. (2020). Realization of artificial synapse and inverter based on oxide electric-double-layer transistor gated by a chitosan biopolymer electrolyte. Semicond. Sci. Technol..

[35] Jiang S., He Y., Liu R., Zhang C., Shi Y., Wan Q. (2021). Synaptic metaplasticity emulation in a freestanding oxide-based neuromorphic transistor with dual in-plane gates. J. Phys. D Appl. Phys..

[36] Woranuch S., Yoksan R. (2013). Preparation, characterization and antioxidant property of water-soluble ferulic acid grafted chitosan. Carbohydr. Polym..

[37] Wu C.T., Lee Y.J., Hsueh F.K., Sung P.J., Cho T.C., Current M.I., Chao T.S. (2014). Characterization of ultra-thin Ni silicide film by two-step low temperature microwave anneal. ECS J. Solid State Sci. Technol..

[38] Bhaskaran M., Sriram S., Perova T.S., Ermakov V., Thorogood G.J., Short K.T., Holland A.S. (2009). In situ micro-raman analysis and X-ray diffraction of nickel silicide thin films on silicon. Micron.

[39] Iwai H., Ohguro T., Ohmi S.I. (2002). NiSi salicide technology for scaled CMOS. Microelectron. Eng..

[40] Bennett K., Lala P.K., Busaba F. (1997). Off-Line Testing for Bridge Faults in CMOS Domino Logic Circuits. NASA University Research Centers Technical Advances in Education, Aeronautics, Space, Autonomy, Earth and Environment.

[41] Byon K., Tham D., Fischer J.E., Johnson A.T. (2007). Systematic study of contact annealing: Ambipolar silicon nanowire transistor with improved performance. Appl. Phys. Lett..

[42] Kim J.B., Fuentes-Hernandez C., Kim S.J., Potscavage W.J., Choi S., Kippelen B. (2010). Ambipolar thin-film transistors with a co-planar channel geometry. Org. Electron..

[43] Wang H., Wang J., Yan X., Shi J., Tian H., Geng Y., Yan D. (2006). Ambipolar organic field-effect transistors with air stability, high mobility, and balanced transport. Appl. Phys. Lett..

[44] Schießl S.P., Fröhlich N., Held M., Gannott F., Schweiger M., Forster M., Scherf U., Zaumseil J. (2015). Polymer-sorted semiconducting carbon nanotube networks for high-performance ambipolar field-effect transistors. ACS Appl. Mater. Interfaces.

[45] Zhou J., Liu Y., Shi Y., Wan Q. (2014). Solution-processed chitosan-gated IZO-based transistors for mimicking synaptic plasticity. IEEE Electron. Device Lett..

[46] Yin C., Li Y., Wang J., Wang X., Yang Y., Ren T.L. (2016). Carbon nanotube transistor with short-term memory. Tsinghua Sci. Technol..

[47] Huang H.Y., Ge C., Zhang Q.H., Liu C.X., Du J.Y., Li J.K., Wang C., Gu L., Yang G.Z., Jin K.J. (2019). Electrolyte-gated synaptic transistor with oxygen ions. Adv. Funct. Mater..

[48] Guo L.Q., Zhu L.Q., Ding J.N., Huang Y.K. (2015). Paired-pulse facilitation achieved in protonic/electronic hybrid indium gallium zinc oxide synaptic transistors. AIP Adv..

[49] Guo L., Wan Q., Wan C., Zhu L., Shi Y. (2013). Short-term memory to long-term memory transition mimicked in IZO homojunction synaptic transistors. IEEE Electron. Device Lett..

[50] Veletić M., Mesiti F., Floor P.A., Balasingham I. Communication Theory Aspects of Synaptic Transmission. Proceedings of the 2015 IEEE International Conference on Communications (ICC).

[51] Lai Q., Zhang L., Li Z., Stickle W.F., Williams R.S., Chen Y. (2010). Ionic/electronic hybrid materials integrated in a synaptic transistor with signal processing and learning functions. Adv. Mater..

[52] Li H.K., Chen T.P., Liu P., Hu S.G., Liu Y., Zhang Q., Lee P.S. (2016). A light-stimulated synaptic transistor with synaptic plasticity and memory functions based on InGaZnO_x_–Al_2_O_3_ thin film structure. J. Appl. Phys..

[53] Lan S., Zhong J., Chen J., He W., He L., Yu R., Chen G., Chen H. (2021). An optoelectronic synaptic transistor with efficient dual modulation by light illumination. J. Mater. Chem. C.

[54] Zucker R.S., Regehr W.G. (2002). Short-term synaptic plasticity. Annu. Rev. Physiol..

[55] Kim S., Choi B., Lim M., Kim Y., Kim H.D., Choi S.J. (2018). Synaptic device network architecture with feature extraction for unsupervised image classification. Small.

[56] Kang D.H., Kim J.H., Oh S., Park H.Y., Dugasani S.R., Kang B.S., Choi C., Choi R., Lee S., Park S.H. (2019). A neuromorphic device implemented on a salmon-DNA electrolyte and its application to artificial neural networks. Adv. Sci..

